# Telehealth Use to Address Cardiovascular Disease and Hypertension in the United States: A Systematic Review and Meta-Analysis, 2011–2021

**DOI:** 10.1089/tmr.2023.0011

**Published:** 2023-05-15

**Authors:** Tiara N. Jackson, Meera Sreedhara, Myles Bostic, Michelle Spafford, Shena Popat, Kincaid Lowe Beasley, Julia Jordan, Roy Ahn

**Affiliations:** ^1^ NORC at the University of Chicago, Chicago, Illinois, USA.; ^2^ Centers for Disease Control and Prevention, Division for Heart Disease and Stroke Prevention, Atlanta, Georgia, USA.; ^3^ Cherokee Nation Operational Solutions, Tulsa, Oklahoma, USA.; ^4^ Veritas Management Group, Inc., Atlanta, Georgia, USA.

**Keywords:** telehealth, cardiovascular disease, hypertension, blood pressure, health equity

## Abstract

**Background:**

The use of telehealth for the management and treatment of hypertension and cardiovascular disease (CVD) has increased across the United States (U.S.), especially during the COVID-19 pandemic. Telehealth has the potential to reduce barriers to accessing health care and improve clinical outcomes. However, implementation, outcomes, and health equity implications related to these strategies are not well understood. The purpose of this review was to identify how telehealth is being used by U.S. health care professionals and health systems to manage hypertension and CVD and to describe the impact these telehealth strategies have on hypertension and CVD outcomes, with a special focus on social determinants of health and health disparities.

**Methods:**

This study comprised a narrative review of the literature and meta-analyses. The meta-analyses included articles with intervention and control groups to examine the impact of telehealth interventions on changes to select patient outcomes, including systolic and diastolic blood pressure. A total of 38 U.S.-based interventions were included in the narrative review, with 14 yielding data eligible for the meta-analyses.

**Results:**

The telehealth interventions reviewed were used to treat patients with hypertension, heart failure, and stroke, with most interventions employing a team-based care approach. These interventions utilized the expertise of physicians, nurses, pharmacists, and other health care professionals to collaborate on patient decisions and provide direct care. Among the 38 interventions reviewed, 26 interventions utilized remote patient monitoring (RPM) devices mostly for blood pressure monitoring. Half the interventions used a combination of strategies (e.g., videoconferencing and RPM). Patients using telehealth saw significant improvements in clinical outcomes such as blood pressure control, which were comparable to patients receiving in-person care. In contrast, the outcomes related to hospitalizations were mixed. There were also significant decreases in all-cause mortality when compared to usual care. No study explicitly focused on addressing social determinants of health or health disparities through telehealth for hypertension or CVD.

**Conclusions:**

Telehealth appears to be comparable to traditional in-person care for managing blood pressure and CVD and may be seen as a complement to existing care options for some patients. Telehealth can also support team-based care delivery and may benefit patients and health care professionals by increasing opportunities for communication, engagement, and monitoring outside a clinical setting.

## Introduction

Cardiovascular disease (CVD), defined as conditions that affect the heart or blood vessels, includes, but is not limited to coronary artery disease, myocardial infarction (heart attack), heart failure, and stroke.^1^ Heart disease is the leading cause of death in the United States (U.S.) and stroke is the fifth leading cause of death—rankings that persisted during the COVID-19 pandemic.^2^ Nearly half the American adult population lives with hypertension, a key risk factor for CVD.^3^ In addition, disparities based on race, socioeconomic status, and insurance status exist for CVD and its risk factors.^4^ Barriers such as inadequate or lack of health insurance coverage, limited access to health care services, and inability to afford out-of-pocket costs can affect U.S. adults' ability to manage their condition.^5^

Telehealth, defined by the Centers for Disease Control and Prevention (CDC) as health care accessed through technology (including mobile phones, smart devices, and computers), has emerged as a well-established approach to address some barriers to accessing health care.^6^ A range of telehealth strategies are commonly used for managing CVD and its risk factors. Remote Patient Monitoring (RPM) represents one such strategy where patients track and submit their blood pressure and other vital signs to health care professionals through an electronic device or through a patient portal.^6^ Synchronous videoconference or teleconference visits represent a different strategy that allows both the caregiver and patient to attend medical visits together irrespective of location through audio-video or audio-only communications.^6^

Telestroke, or technology-based care for strokes, and home-based cardiac rehabilitation may be offered through videoconferencing or teleconferencing. Alternatively, mobile health technologies (mHealth) are health-related applications on mobile phones or other “smart devices” (e.g., apps, specialty websites, or smartwatches) and can include appointment and medication reminders through text messages.^7^ Health care professionals may choose to implement a combination of telehealth strategies to manage CVD and its risk factors and supplement services through text messaging (e.g., medication reminders or lifestyle coaching),^8^ synchronous teleconferencing or videoconferencing with pharmacists and other health care professionals,^9–16^ and interactive voice response (IVR) systems.^17^

A gap in the literature exists, summarizing the use of these common categories of telehealth strategy and impact on CVD and hypertension outcomes specifically within the United States, and including during the COVID-19 pandemic. Many of the systematic reviews on telehealth include studies conducted internationally^7,18–23^ and few described telehealth interventions conducted during the COVID-19 pandemic.^24^ These previously published systematic reviews either focused on a single health condition^18^ or telehealth strategy,^7,21,22^ or more generally assessed telehealth across conditions,^19,22–25^ some of which included cardiovascular and noncardiovascular conditions.

Telehealth utilization across medical specialties and conditions greatly expanded in the United States during the pandemic as health care professionals were encouraged to adopt virtual care strategies to continue care, while preventing the spread of COVID-19. The American Medical Association estimated that telehealth visits increased to 35 million in the second quarter of 2020 compared to 1.4 million visits in the previous quarter.^26^ Although telehealth has the potential to expand access to care for communities who are underserved by geographic location, there are concerns that telehealth may exacerbate health disparities especially for 25% of U.S. adults who lack broadband (or high speed) internet and those without adequate access to technology.^19,27^

Other barriers beyond access to technology or internet include limited English proficiency and digital literacy. Furthermore, many health systems may face challenges procuring telehealth equipment and software, and identifying staff to support telehealth services, such as RPM.^28,29^ With the exception of telestroke, the lack of strong evidence for telehealth for hypertension and CVD management among U.S. populations and specifically for patients at highest risk for CVD and hypertension,^25^ combined with the rapid expansion of telehealth by health systems during the COVID-19 pandemic, necessitates an assessment of telehealth strategies for the management of CVD and its risk factors.^26^

As telehealth utilization expands, the considerations of those disproportionately affected by cardiovascular conditions need to be prioritized. Currently, there is sparse literature summarizing telehealth outreach, impacts on access to care, health equity, social determinants of health, and outcomes related to hypertension and CVD for populations disproportionately affected by cardiovascular conditions.^25^ More evidence is needed to support the expansion of telehealth services to manage CVD-related conditions and hypertension. In 2021, researchers from CDC's Division for Heart Disease and Stroke Prevention and NORC at the University of Chicago (NORC) conducted a literature review of telehealth interventions to address hypertension and CVD management and control. This article summarizes current evidence on telehealth for hypertension and CVD management.

The purpose was to identify how telehealth is being used by U.S. health care professionals to manage hypertension and CVD and describe the impact these telehealth strategies have on hypertension and CVD outcomes, with a focus on social determinants of health and health disparities. The review included a narrative review of the literature and meta-analyses to examine the impact of specific U.S.-based hypertension and CVD management telehealth interventions on selected outcomes amenable to analysis, including changes in systolic blood pressure (SBP) and diastolic blood pressure (DBP), all-cause mortality, and all-cause hospitalizations.

## Methods

In December 2021, we manually searched PubMed/Medline for articles published in English and studies conducted in the United States between 2011 and 2021. We restricted the search to U.S.-based interventions due to unique telehealth regulatory and reimbursement mechanisms and implementation factors in the United States.^19^ To systematically identify articles that aligned with the review's purpose, we created a search string with every possible combination of terms. A full list of search terms is included in Table 1. The following categories describe the types of terms included in the search: telehealth strategies (e.g., telehealth, videoconferencing), CVD, hypertension and related interventions (e.g., hypertension, cardiovascular disease), evaluation (e.g., evaluation, assessment), and other terms. Other terms included COVID-19, social determinants of health, and health disparities, but not health equity because the latter term restricted findings during a preliminary test of the search string.

**Table 1.** Literature Review Search Terms

Search category	Terms
Telehealth strategies	Telehealth, telemedicine, videoconferencing, store-and-forward, remote patient monitoring, mHealth, mobile, text messaging, audio only, synchronous telemedicine, asynchronous telemedicine
CVD, hypertension, and related interventions	Hypertension, cardiovascular disease, blood pressure, cholesterol, pre-hypertension, heart failure, stroke, cardiac rehabilitation, medication therapy management, telestroke
Evaluation	Evaluation, assessment, medication adherence, outcome, implementation effectiveness, sustainability, cost effectiveness, program development, evaluation study, program evaluation, Enhanced Evaluability Assessment (EEA), Systematic Screening and Assessment (SSA), Consolidated Framework for Implementation Research (CFIR), Health Services Availability
Other	COVID-19, health disparities, social determinants of health

CVD, cardiovascular disease; mHealth, mobile health technologies.

**FIG. 1.**
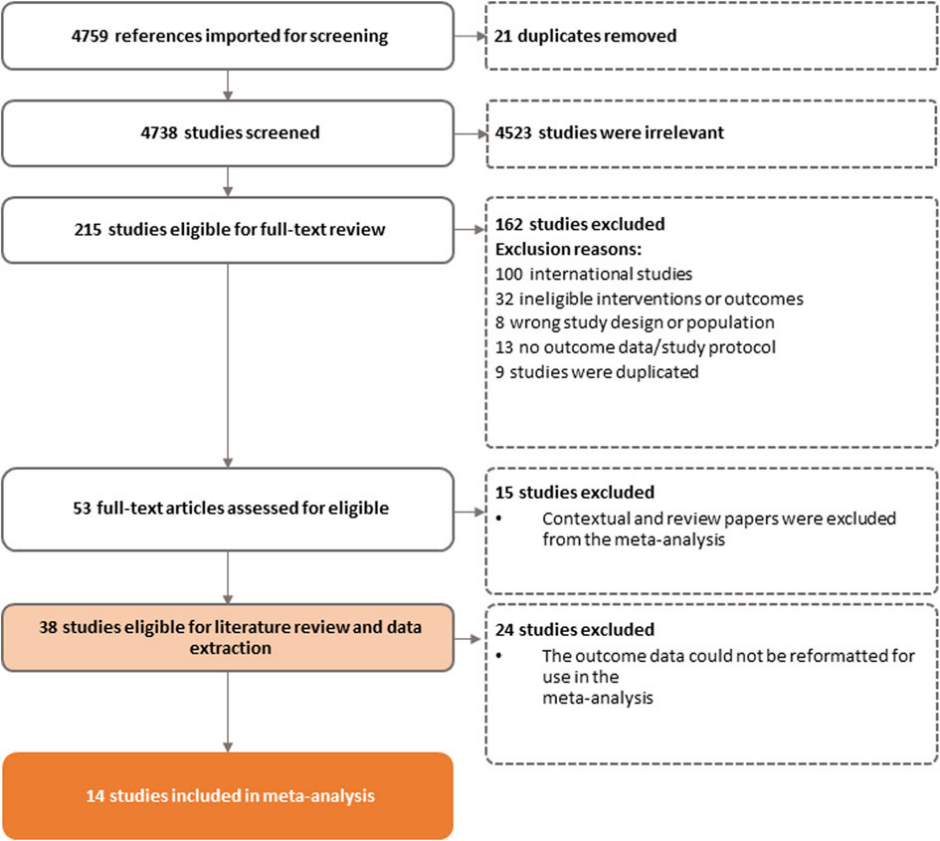
PRISMA diagram for peer-reviewed articles screened.

All potentially relevant peer-reviewed articles were uploaded to Covidence (Veritas Health Innovation, Melbourne, Australia), an online literature review management program, for title and abstract review, full-text review, and data extraction. Based on our initial search criteria (Table 2), we identified a total of 4759 articles, of which 21 were duplicate articles (Fig. 1). After the duplicate articles were removed, at least two research team members reviewed the title and abstract of each article to determine if articles met the *a priori* criteria listed in Table 2.

**Table 2.** Literature Review Selection Criteria for the Review

Criteria category	Specifications
Publication date	2011–2021
Population	Adult patients (18 years of age or older) with a hypertension diagnosis Patients with diabetes and/or eclampsia are excluded
Study design and outcomes	U.S.-based studies
Intervention description	Clinically based studies only Web- or telephone-based communications Must have two-way communication between the patient and health care professional Lifestyle interventions are excluded

A total of 215 articles met these criteria and were included in the full-text review, where two team members individually reviewed the articles. Throughout the review process, if there were disagreements about inclusion, a third team member resolved the conflict. During the full-text review, the team confirmed that each article met all criteria. A total of 38 articles about specific telehealth interventions, which have been implemented across the country, met the criteria for inclusion into the literature review (see Fig. 1 for the PRISMA diagram and Table 3 for a description of the studies).

**Table 3.** Relevant Studies Included in this Literature Review

Study ID	Intervention type	Health condition	Primary outcome(s)	Study description
Angellotti et al.^8^	RPM and Telehealth	Cardiometabolic diseases (incl. hypertension)	Telehealth feasibility and acceptability	Participants received personalized text messages of recommendations and motivation regarding diet, exercise, and medication adherence. A subset of participants was additionally asked to measure and report BP twice a day using Bluetooth-enabled BP monitor and the iHealth MyVitals app.
Baidwan et al.^48^	Telehealth	Hypertension	BP	An organization-level analysis was conducted using the Uniform Data System administrative database to assess the use of telehealth in community health centers for patients with hypertension. The nature and scope of telehealth included were not specified.
Bekelman et al.^35^	RPM	Heart failure	Mortality	Participants received a multicomponent intervention to improve health status, which included RPM and patient self-care support (i.e., medication reminders, health education), collaborative care disease management, and screening and treatment of depression.
Benson et al.^36^	RPM with Teleconferencing	Hypertension	BP; medication adherence	The HeartBeat Connections program, a teleconferencing-based health coaching program, was administered to participants. RDNs and RNs provided one 20-min call per month to discuss medication management and biometric and lifestyle CVD risk factors. Participants additionally received a physical handbook on CVD prevention. The intervention was provided as a complement to usual care.
Blum and Gottlieb^63^	RPM	Heart failure	Hospitalization; mortality	The RPM protocol consisted of daily BP, heart rate, weights, and 15-sec heart rhythm strip readings that were transmitted wirelessly to the patient's file. Nurse practitioners followed up with patients whose readings were outside of individually assigned parameters.
Bosworth et al.^42^	Remote Monitoring and Telehealth	Hypertension	BP	Participants received one of three interventions: (1) physician- and nurse-administered medication management, (2) nurse-administered behavioral management, or (3) a combination of the first two. Intervention activities included health behavior education and modifications, and medication adjustment. Participants were provided with a wireless home BP monitor and telemedicine device to record their BP once every other day. Teleconferencing could be triggered based on the participants' BP metrics measurements.
Bowles et al.^64^	RPM with Videoconferencing	Heart failure	Hospitalization	Participants measured predetermined biometrics at home, which were automatically transmitted to their care team and monitored by a nurse. Readings outside of normal range were reported to the care team to determine changes in the treatment plan. Participants additionally received six videoconferencing visits and four in-person home visits from a nurse.
Clark et al.^59^	RPM with Videoconferencing	Hypertension	BP	A prospective cohort study comprising of patients with uncontrolled hypertension. Patients in the intervention group received a BP cuff monitor and tablet. Participants were required to transmit BP readings twice daily. Patients had live video BP review appointments with a pharmacist every 3 weeks.
Choudhry et al.^33^	RPM	Hypertension	BP; medication adherence	Participants received a multicomponent intervention to address medication adherence. The intervention comprised tailored teleconferencing visits with a clinical pharmacist, mailed progress reports, and tailored medication adherence strategies, which could include text messaging and pill boxes, follow-up consultations, and structured reports sent to the participant's primary care physician with care coordination recommendations.
Dalouk et al.^49^	Videoconferencing	Heart failure	Mortality	Participants were enrolled in a Telemedicine Videoconferencing Clinic, which included an unspecified number of video visits with a physician or Nurse Practitioner. Participants were additionally enrolled in RPM programs as available.
Davis et al.^37^	RPM	Heart failure	Hospitalization	Participants recorded daily symptoms and received print educational materials on symptom management. Home visits from a care team were prompted by either care team review of recorded symptoms or by request.
de Peralta et al.^51^	Video and Teleconferencing	Heart failure	Hospitalization	Participants received consultation through phone or video-to-home virtual care from a Nurse Practitioner. Participants were provided with care team recommendations following the virtual visit.
Fisher et al.^9^	RPM	Hypertension	BP	Participants received a Bluetooth-enabled home BP device that automatically transmitted BP readings to their care team. Medication could be adjusted by teleconferencing on a biweekly basis dependent on participant readings. Nurse practitioners and pharmacists managed the participants.
Friedberg et al.^44^	Teleconferencing	Hypertension	Assessment of patient reach/feasibility	Participants received a monthly behavioral intervention aimed at improving treatment adherence through telephone. Interventions were grounded within the Stages of Change and Transtheoretical Models.
Idris et al.^10^	RPM with Videoconferencing	Heart failure	Hospitalization; mortality	Participants in the Health Connect system intervention received weekly videoconferencing calls, as well as daily RPM of BP, weight, oxygen saturation, and heart rate. Videoconferencing calls covered topics such as medication regiments, concerns with regard to care, office visits, and any additional care question that the participant may have. Patient data were reviewed by a physician or nurse with expertise in cardiology. The cardiologist was consulted to adjust medication or recommend for an office visit when parameters were outside of a normal range.
Kao et al.^11^	RPM	Heart failure	Hospitalization; mortality	The Health Buddy Program combined telehealth and care management. Participants were provided with a Health Buddy Device within their home to record daily vital signs and health status information. Participants received automated educational feedback and were triaged into low-, medium-, and high-risk categories based on information provided. Next step care was subsequently determined based on risk level.
Kothapalli et al.^60^	RPM	Cardiovascular disease	BP	This clinical trial utilized a web-based telemedicine system. Participants were asked to report BP, daily steps, weight, and cigarette usage once a week.
Lakshminarayan et al.^61^	RPM	Hypertension (stroke)	Medication adherence	Participants were given a wireless BP monitor to measure their BP daily. Medications were managed and adjusted accordingly on a biweekly basis by a physician and pharmacist.
Litke et al.^45^	Video and Teleconferencing	Hypertension	BP	The intervention aimed to improve access to care for rural veterans through a combined telehealth and CPS program. Participants received medication management videoconferencing and teleconferencing visits with a CPS.
Magid et al.^17^	RPM and Teleconferencing	Hypertension	BP; medication adherence	The multimodal intervention included home BP monitoring and reporting three to four times a week to an IVR telephone system, as well as patient education. Participants were managed by a clinical pharmacist and additionally had the opportunity to request a teleconferencing visit with the clinical pharmacist or to receive educational information.
Mallow et al.^34^	RPM	Chronic conditions (incl. hypertension)	BP	Participants recorded BP, weight, and blood glucose on a tablet and Bluetooth-enabled self-monitoring device. Participants were also provided access to mL SMART, a web-based platform with asynchronous and synchronous features, some of which include videoconferencing, messaging portal, health education, reminders and notifications of medications, and access to readings.
Margolis et al.^38^	RPM with Teleconferencing	Hypertension	BP	Participants measured their BP with home BP telemonitors. Metrics were reviewed by pharmacists, who adjusted medications accordingly and provided information regarding medication adherence and lifestyle modifications through teleconferencing.
Milani et al.^14^	Remote Monitoring and Telehealth with Teleconferencing	Hypertension	BP	Participants were asked to provide weekly BP readings, which were automatically transmitted to their EHR. Pharmacists and health coaches delivered education, recommendations, and medication management through telephone calls. Participants were additionally directed to a hypertension management educational website.
O'Connor et al.^39^	RPM	Heart failure	Hospitalization	Grounded within the Transitional Care Model, the telehealth intervention employed a wireless tablet-based system to collect participant biometrics, including BP, heart rate, weight, and blood oxygenation. Participants additionally received instructional videos.
Ong et al.^65^	RPM with Teleconferencing	Heart failure	Hospitalization; mortality	The Better Effectiveness After Transition—Heart Failure study employed an intervention to address care transition in heart failure patients. RNs health coaching teleconferencing with participants. Participants additionally recorded their BP, heart rate, weight, and symptoms daily through a Bluetooth-enabled device, which were reviewed by RNs.
Ovbiagele et al.^55^	RPM	Stroke	BP; medication adherence	Participants received an electronic medication tray and a Bluetooth-enabled BP monitor. They were additionally provided with weekly tailored email reports based on BP readings and medication adherence rates.
Pekmezaris et al.^40^	RPM with Videoconferencing	Heart failure	Hospitalization	Participants were provided with an in-home American TeleCare video patient station, which included a built-in BP monitor and stethoscope. Nurses conducted videoconferencing visits where they guided participants in measuring their weight, BP, and heart rate.
Piette et al.^56^	RPM with Teleconferencing	Heart failure	Medication adherence	Participants received weekly IVR teleconferencing about self-management and health over the course of 12 months. Participants were additionally asked to identify a “CarePartner” who was external to their household. The predetermined CarePartner received automated emails with suggestions for how to support disease care for the participant.
Polgreen et al.^57^	Remote Monitoring and Telehealth	Hypertension	BP	Targeting rural areas, participants were asked to provide 3 days of BP readings for each month of the intervention. Pharmacists certified as hypertension clinicians provided education and recommendations to participants through telephone, email, and text message.
Ralston et al.^15^	Teleconferencing	Hypertension	BP; medication adherence	Clinical pharmacists met with participants at the beginning of the intervention through teleconferencing to establish an action plan. Clinical pharmacists continued communication by messaging at least once every 2 weeks for the first 2 months, monthly in the fourth through sixth months, and every 3 months for the rest of the study. Participants were additionally provided home BP monitors.
Rosen et al.^41^	RPM with Videoconferencing	Heart failure	Hospitalization	Participants were asked to complete daily check-ins of health status (i.e., medication adherence, health concerns) through a telehealth platform. Participants additionally received weekly videoconferencing from a social worker trained in heart failure coaching.
Simpson et al.^53^	Videoconferencing (Telestroke)	Stroke	Mortality	This population-level study analyzed billing data and assessed the impact of exposure to the state telestroke network within patients suffering acute ischemic stroke.
Sobhani et al.^52^	Videoconferencing (Telestroke)	Stroke	Hospitalization; mortality	Hub vascular neurologists provided consultation to network spoke facilities for all patients presenting to the ED with stroke.
Taylor et al.^47^	Videoconferencing	Hypertension	BP	Physicians and nurse practitioners conducted videoconferencing appointments with patients. Topics discussed included lifestyle modifications and medication adherence.
Updike et al.^16^	RPM with Videoconferencing	Hypertension	BP	In this intervention, participants received a Bluetooth-enabled BP monitor and were asked to provide daily BP readings. Participants additionally received weekly videoconferencing appointments with pharmacists to discuss their BP readings, goals, and lifestyle modifications.
Wakefield et al.^43^	RPM with Teleconferencing	Hypertension	Medication adherence	Participants were placed into one of three groups: (1) usual care (control group); (2) low intensity; and (3) high intensity. Both intervention groups manually entered BP and blood glucose into an in-home telehealth device. The high-intensity group received questions and informational tips regarding lifestyle modifications and medications through teleconferencing. The low-intensity group received the questions, but no informational tips. All participants were managed by nurses.
Yuan et al.^50^	Video and teleconferencing	Heart failure	Hospitalization	Yuan et al. conducted a visit-level analysis comparing in-person, video-based, and telephone-based ambulatory cardiology visits for heart failure.
Zha et al.^58^	RPM	Hypertension	BP	This randomized control trial provided participants with a Bluetooth-enabled BP monitor that paired with the iHealth MyVitals app, which provided feedback to participants. Community health center nurses, community health workers, and other health care professionals were able to remotely monitor participants' BP.

BP, blood pressure; CPS, clinical pharmacy specialists; ED, emergency department; EHR, electronic health record; IVR, interactive voice response; RDNs, registered dietitians and nutritionists; RPM, remote patient monitoring; RNs, registered nurses.

After the full-text review, we evaluated how each study reported their outcomes (e.g., differences among groups, raw or adjusted values) and if the outcomes could be synthesized with the other studies to conduct a meta-analysis. After reviewing the available data, we selected the following outcomes for the meta-analysis: change in SBP and DBP for all conditions, all-cause hospital admissions, and all-cause mortality. All-cause hospital admissions and all-cause mortality were analyzed in the meta-analysis instead of cause-specific outcomes because of the variation in how cause-specific measures were analyzed and reported.

Inclusion criteria for the meta-analysis consisted of (1) a clear intervention and control group; (2) outcome data for *both* the intervention and control groups; and (3) comparable outcome measures (group means and standard errors for blood pressure and proportions or raw values for hospitalizations and mortality). Overall, 14 of the 38 articles describing telehealth interventions met the inclusion criteria for the meta-analysis (Table 4). Relevant information was extracted from each of these articles, including methodological and statistical details (i.e., statistical analysis process, sample size, quantitative outcomes, etc.).

**Table 4.** Evaluation Characteristics of Studies in the Meta-analysis (*n* = 14)

Study ID	Evaluation design	Sample size	Mean SBP change (SD)	Mean DBP change (SD)	% hospitalizations	% mortality
Bekelman et al.^35^	Randomized Control Trial	Treatment: 187 Control: 197	—	—	Treatment: 29% Control: 29%	Treatment: 4% Control: 10%
Benson et al.^36^	Quasi-Experimental	Treatment: 326 Comparison: 702	Treatment: 0.1 (1.4) Comparison: −0.9 (1.3)	Treatment: −1.3 (0.9) Comparison: −2.3 (0.8)	—	—
Blum and Gottlieb^63^	Randomized Control Trial	Treatment: 93 Control: 182	—	—	Treatment: 31% Control: 38%	—
Bowles et al.^64^	Randomized Control Trial	Treatment: 101 Control: 116	—	—	Treatment: 16% Control: 19%	—
Clark et al.^59^	Quasi-experimental	Treatment: 118 Comparison: 871	Treatment: −14.1 (15.0) Comparison: −7.3 (20.3)	Treatment: −7.9 (8.6) Comparison: −2.4 (12.8)		
Davis et al.^37^ 2015	Quasi-experimental	Treatment: 59 Comparison: 59	—	—	Treatment: 8% Comparison: 17%	—
Idris et al.^10^	Randomized Control Trial	Treatment: 14 Control: 14	—	—	Treatment: 7% Control: 50%	—
Kao et al.^11^	Quasi-experimental	Treatment: 230 Comparison: 230	—	—	—	Treatment: 25% Comparison: 48%
Magid et al.^17^	Randomized Control Trial	Treatment: 138 Control: 145	Treatment: −13.1 (20.4) Control: −7.1 (16.6)	Treatment: −6.5 (11.7) Control: −4.2 (10.4)	—	—
Margolis et al.^38^	Randomized Control Trial	Treatment: 188 Control: 182	Treatment: −21.3 (20.3) Control: −14.7 (20.0)	Treatment: −9.3 (16.4) Control: −6.4 (16.5)	—	—
Ong et al.^65^	Randomized Control Trial	Treatment: 715 Control: 722	—	—	Treatment: 23% Control: 22%	Treatment: 3% Control: 5%
Pekmezaris et al.^40^	Randomized Control Trial	Treatment: 83 Control: 85	—	—	Treatment: 30% Control: 29%	—
Simpson et al.^53^	Quasi-experimental	Treatment: 27,042 Comparison: 12,322	—	—	—	Treatment: 6% Comparison: 7%
Sobhani et al.^52^	Quasi-experimental	Treatment: 55 Comparison: 87	—	—	Treatment: 9% Comparison: 17%	Treatment: 2% Comparison: 2%

DBP, diastolic blood pressure; SBP, systolic blood pressure; SD, standard deviation.

We made two assumptions to convert data into a consistent and usable format (e.g., means and standard deviations) to conduct the meta-analysis because incorrect assumptions can cause inaccuracy in the impact estimates for individual studies and pooled estimates. After standardizing the parameters for the included studies, we conducted our analysis using Stata 16^30^ and ran random-effects models using the DerSimonian and Laird method.^31,32^ The first assumption was made about random-effect models and we assumed that studies included in the meta-analysis were a random sample of the distribution of effects and allow the true effect to vary from study to study. This method weighs studies based on the inverse of the sum of the variance estimated between studies and the individual sampling variance.

The next assumption made was that all included studies had enough in common to be incorporated in the meta-analysis for synthesis. Although studies were similar, they may still have varied effect estimates due to factors such as program setting, program feature, patient population, study design, and analytic method. Therefore, we used random-effects meta-analysis to account for variation (heterogeneity) in effect estimates across studies by these factors.

## Results

The 38 articles identified during the literature review (Table 5) highlight the following telehealth strategies to manage hypertension and CVD: RPM; synchronous teleconferencing and/or videoconferencing (including telestroke); and a combination of telehealth strategies, which often includes mHealth.

**Table 5.** Key Intervention Characteristics (*n* = 38)

Health condition	Primary intervention type	Primary outcomes
Hypertension (*n* = 21)	RPM (*n* = 15) Teleconferencing and/or videoconferencing (*n* = 6)	BP (*n* = 17) Medication adherence (*n* = 7)
Heart Failure (*n* = 14)	RPM (*n* = 11) Teleconferencing and/or videoconferencing (*n* = 3)	Medication adherence (*n* = 1) Hospitalizations (*n* = 11) Mortality (*n* = 6)
Stroke (*n* = 3)	Telestroke (*n* = 3)	BP (*n* = 1) Hospitalizations (*n* = 1) Mortality (*n* = 2)

This section describes characteristics of the specific telehealth interventions that were included in the literature review and the findings related to social determinants of health and health disparities. The final section summarizes the corresponding outcomes for patients, health care professionals, and health systems.

### Description of intervention characteristics

#### Remote patient monitoring

Twenty-six of the 38 intervention studies included in this review focused on the use of RPM. All RPM interventions included in this review contained bidirectional communication channels, or the ability for both patients and health care professionals to send and receive information. Collectively, the focus of RPM in the studies included hypertension (*n* = 15), chronic heart failure (*n* = 11), and chronic obstructive pulmonary disease (COPD) (*n* = 1; COPD was examined with heart failure in one study). The devices or tools to measure biometric outcomes were maintained in the patients' homes.^11^

These data were transmitted in real time to a patient's electronic health record (EHR) or to a remote monitoring care provider (either at a medical facility or off-site through a third-party vendor). Some patients manually entered their vital measurements into a web-based portal or through an IVR system. In RPM, communication from the health care professionals varies: some engaged only when abnormal values were detected, while others engaged with patients at regularly scheduled time intervals. Some RPM interventions included secure messaging threads, in addition to telephone follow-up calls.^15,33,34^ Regardless of communication frequency, these follow-up visits were often used as an opportunity to assess patient adherence with their treatment plan and provide behavioral counseling.

While physicians played a role in most RPM programs, these programs were not commonly directly led by physicians.^16,17,33,35–43^ Among the interventions reviewed, RPM implementation included clinical staff such as advanced practice providers, pharmacists, nurses, and dieticians as well as nonclinical staff such as lay navigators and social workers. Most RPM interventions used a team-based approach with a nurse and/or pharmacist who served as a coordinator between the patient and the care team.^17,33,35,36,38,40,42,43^ Physicians were updated about patients, typically through the EHR. When abnormal values were detected or changes to a treatment plan were needed, physicians were contacted to make necessary changes.

#### Synchronous videoconferencing and teleconferencing services

Twelve of the intervention studies used videoconferencing or teleconferencing services to interact with health care professionals (e.g., physicians, pharmacists, nurses, psychologists). These interventions focused on managing and treating hypertension (*n* = 7),^15,17,44–48^ heart failure (*n* = 3),^49–51^ and stroke (*n* = 2).^52,53^ These health care professionals were affiliated with primary care clinics, hospitals, and cardiology specialty groups. Medication management was commonly offered through synchronous teleconferencing with pharmacists. Two articles in the review focused on telestroke services.^52,53^ The telestroke care teams consisted of a neurologist, neuroradiologist, neurocritical care specialist, and a neurosurgeon.^54^ These staff were supported by midlevel telestroke staff and nursing personnel during the acute phase (first few hours) of an ischemic stroke.

#### Combination telehealth interventions/strategies

Half (*n* = 19) of the interventions reviewed reported a combination of telehealth strategies to manage hypertension and/or CVDs. Supplemental services included text messaging (e.g., medication reminders or lifestyle coaching),^8^ synchronous teleconferencing or videoconferencing with pharmacists and other health care professionals,^9–16^ and IVR systems.^17^ None of the studies included in our review focused solely on mHealth, whereas nine of the interventions using RPM included one or more mHealth components.^8,14,15,33,34,55–58^

For some health conditions, in-person visits may still be needed; in these cases, telehealth visits supplement in-person care. For example, interventions involving patients with heart failure were sometimes conducted in conjunction with home-based care, which include regular home visits by home health nurses and aides. In a study by Pekmezaris et al.,^40^ recently discharged patients with heart failure were able to be treated with both live and video home health nurse appointments as a follow-up to RPM. The number of in-person visits gradually decreased over time, and by the end of the intervention, in-person visits were conducted only if a nurse deemed the visit necessary.

### Social determinants of health and health disparities

Our review did not find peer-reviewed articles specifically focused on addressing social determinants of health or health disparities through telehealth interventions for hypertension or CVD. One intervention in the review that delivered a telehealth intervention to a diverse study population was the Hypertension Intervention Nurse Telemedicine Study (HINTS).^42^ HINTS pertained to the evaluation of the effect of nurse- and physician-administered telephone-based telemonitoring interventions on blood pressure among patients at the Veterans Affairs Hospital in Durham, NC. Nearly half of the study population comprised African American patients (48%). One-third had a low literacy level (38%) and were employed (35%). Two interventions in the HINTS study significantly improved blood pressure control relative to usual care at 12 months: behavioral management telemonitoring (*p* = 0.03) and medication management telemonitoring (*p* = 0.03), but outcomes were not compared by patient characteristics.

### Outcomes for patients, health care professionals, and health systems

Among the 38 telehealth interventions reviewed, the primary CVD-related risk factors and outcomes targeted were hypertension (*n* = 21), heart failure (*n* = 14), and stroke (*n* = 3). Among these conditions, the primary outcomes of interest varied, but included changes in blood pressure (including blood pressure control), medication adherence, hospitalizations, and all-cause mortality (Table 5). Additional outcomes identified included patient engagement and satisfaction.

Below, we summarize outcomes for these 38 interventions, as well as the outcomes for the subset of articles that were eligible for the meta-analyses.

### Patient outcomes

#### Blood pressure

Eighteen telehealth interventions examined blood pressure as an outcome.^9,13–17,33,34,36,42,45,47,48,55,57–60^ These interventions were effective at helping patients reduce their overall blood pressure and improve control and management of their blood pressure. Studies using RPM with a supplemental telehealth intervention commonly reported reductions in blood pressure at the conclusion of the study. Most studies did not find significant differences in post-intervention blood pressure between treatment and usual care groups. Furthermore, patients utilizing telehealth were able to maintain improvements in blood pressure, with three studies seeing improved outcomes lasting for 12 or more months.^15,33,42^

**FIG. 2.**
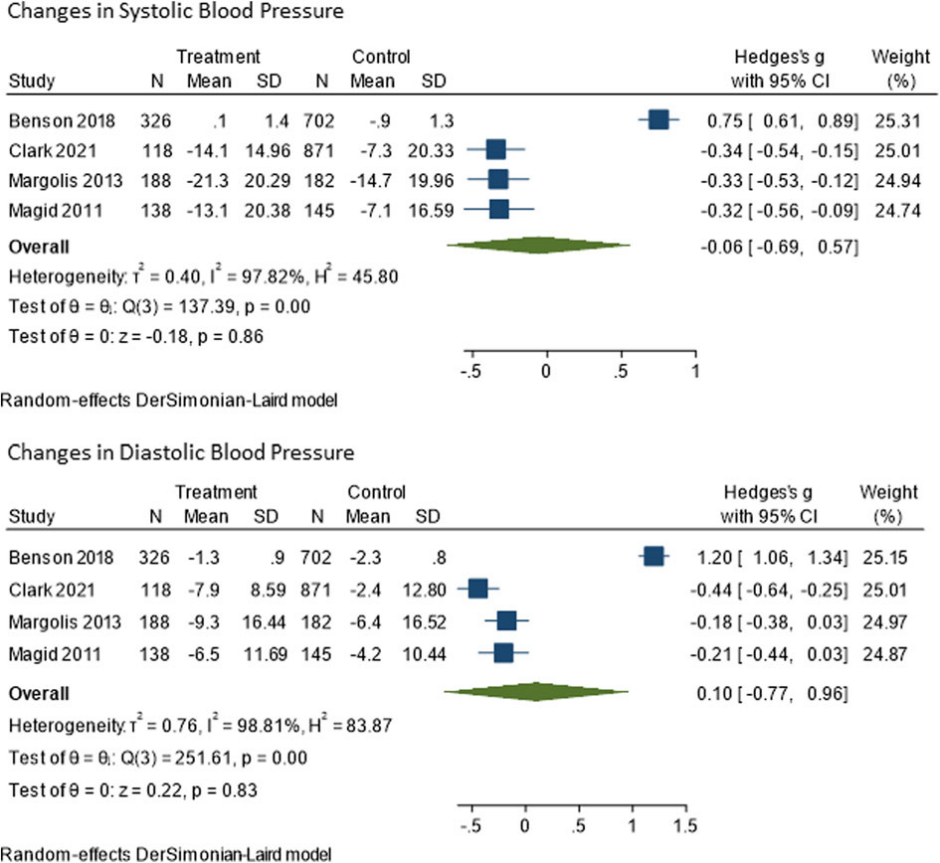
Meta-analysis results for changes in systolic and diastolic blood pressure. CI, confidence interval; SD, standard deviation.

Among studies included in the meta-analysis (*n* = 4), changes in blood pressure were null for the intervention groups (Fig. 2). The difference in SBP from baseline to end of study was 0.06 mmHg (confidence interval [95% CI]: −0.69 to 0.57) less for the treatment group compared to the control group. Change in DBP was 0.10 mmHg (95% CI: −0.77 to 0.96) higher in the treatment group compared to the control group. Both values were not statistically significant.

#### Medication adherence and management

Eight interventions assessed medication adherence.^15,17,33,36,43,55,56,61^ For the studies measuring this outcome, medication adherence was assessed using patient self-report (*n* = 5)^17,36,43,56,61^ and prescription claims data (*n* = 2).^15,33^ One study did not describe how it assessed medication adherence.^55^ Self-reports of medication adherence were collected through telehealth visits, surveys, and a mobile app or patient portal. Engagement in telehealth interventions yielded mixed results for medication adherence, with most interventions not seeing significant differences between treatment and usual care groups. Although, for one study examining the impact of a pharmacist-led telehealth intervention across 14 primary care practice sites,^33^ significant improvements in medication adherence occurred, but did not translate into significant clinical improvements in blood pressure for the treatment group.

Regular interactions with health care professionals through telehealth did result in more responsive adjustments to medication treatment plans. In some studies,^13,15^ this resulted in the treatment group having more medication changes, including changes in dosage and medication intensity (e.g., stronger antihypertensive medicines) added to patients' treatment plans. For two of the studies that saw significant improvements in blood pressure,^13,15^ these changes were mediated by regular use of home blood pressure monitors and adjustments in medication because of results transmitted to pharmacists.

Telehealth, particularly interventions that include monitoring, allowed health care professionals to engage with their patients more often and receive more information about their patients' health status.^62^ These additional interactions and information sharing helped them to understand how a patient was responding to their medicine and adjust as needed. When monitoring was coupled with follow-up calls, patients were able to express challenges to maintaining their medicine regimen such as side effects or cost, allowing the health care team to be more responsive than waiting for traditional follow-up appointments.

To help with medication management and adherence, some interventions utilized pharmacists and social workers as a part of the telehealth intervention. Other interventions, such as that described by Ralston et al.,^15^ utilized decision support tools embedded in the telehealth platform to identify patients needing adjustments to medication regimens, whereas in Rosen et al.,^41^ social workers were trained as health coaches specifically for patients with heart failure, resulting in 96% adherence to treatment protocols.

#### Hospitalizations

Twelve studies in this review assessed all-cause hospitalization rates.^10,11,37,39–41,50–52,63–65^ Hospitalization rates among patients in telehealth interventions focused on hypertension and CVD management were similar to those receiving usual care. The data related to hospitalization were mixed, with some studies finding significant reductions, while others found null results. Two interventions in this review saw significant reductions in hospitalizations among treatment groups. In addition, one study found hospitalizations among patients with heart failure receiving telephone-based care to be higher compared to patients with heart failure receiving video or in-person care.^50^ A study by Davis et al.^37^ saw a reduction in 30-, 90-, and 180-day all-cause hospital readmission rates, but not emergency room visits among patients in the telemonitoring intervention group.

Four studies specifically looked at cardiac-related hospitalization and readmission outcomes (i.e., hospital admissions and readmission due heart failure; emergency department visits and hospitalizations that had an encounter diagnosis of heart failure within 90 days of a cardiology clinic visit for heart failure; and heart failure-related rehospitalizations, hospital days, and emergency department visits). Three out of the four studies found no significant difference in heart failure-related hospitalizations or readmissions between the intervention and control groups. One study found that the intervention group had significantly fewer hospitalizations compared to the control group (*p* = 0.03).^10,36,45,58^

In an intervention to test the feasibility of switching to 100% video telehealth appointments for patients with heart failure during the COVID-19 pandemic, de Peralta et al.^51^ also assessed whether nurses could properly identify patients who needed hospitalization through telehealth. In this intervention, a specialized protocol of assessments, such as checking oxygen levels and surveying the patient for edema, was conducted by a nurse practitioner over a video telehealth appointment. Using decision support tools to review the results of the assessment, patients most at risk for a cardiac-related event were advised to call 911 for assistance. Similarly, Pekmezaris et al.^40^ found that participants with heart failure, who were enrolled in RPM with follow-up video calls, were hospitalized up to 4 days sooner than patients in the usual care group.

**FIG. 3.**
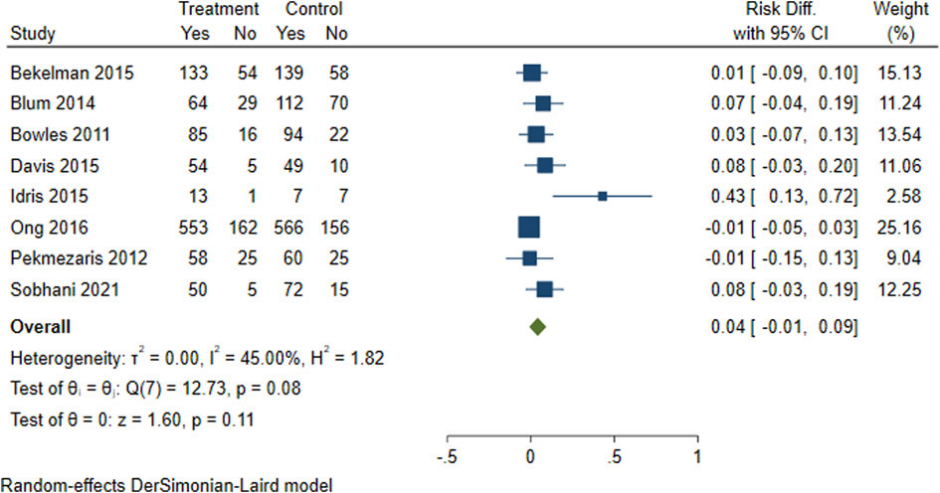
Meta-analysis results for all-cause hospitalizations among patients with stroke and heart failure.

Among the studies included in this meta-analysis that assessed all-cause hospitalization(*n* = 8), the intervention effect on all-cause hospitalizations was also null (Fig. 3).

#### Mortality

Eight interventions included in the review assessed mortality, which were conducted among heart failure (*n* = 6)^10,11,35,49,63,65^ and stroke (*n* = 2)^52,53^ patients. As with hospitalizations, mortality from all causes was assessed by these studies. The impact of telehealth interventions on mortality was mixed, with three studies yielding significant results. These three studies focused on patients with heart failure (*n* = 2) and stroke (*n* = 1). It is unclear why these interventions improved mortality-related outcomes. No study specifically addressed cardiac-related mortality.

**FIG. 4.**
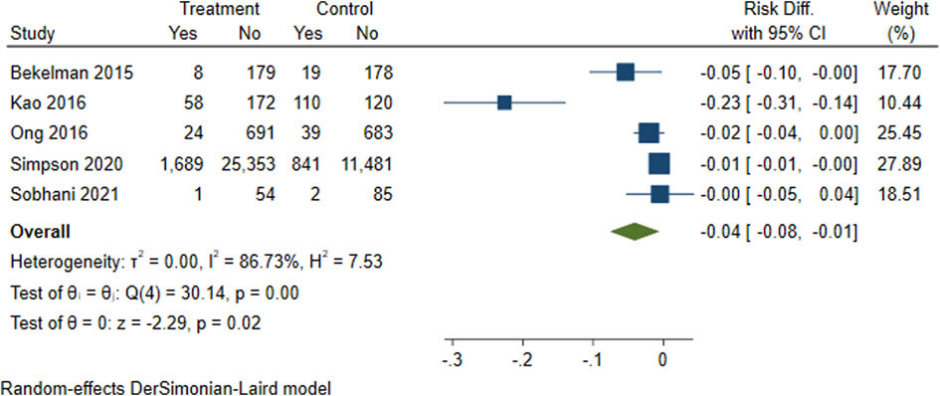
Meta-analysis results for all-cause mortality among patients with heart failure or stroke.

Among the studies included in the meta-analysis (*n* = 5), patients in the treatment group saw reductions in all-cause mortality ranging from 0% to 23% when compared to the control group (Fig. 4). Patients with heart failure receiving remote monitoring and patients who received telestroke services had an overall statistically significant 4% reduction in all-cause mortality (95% CI −0.08 to −0.1). A reduction in all-cause mortality was the only statistically significant outcome observed in the meta-analysis.

#### Patient engagement and satisfaction

Several studies in this review have indicated that patient satisfaction with telehealth is high, with some patients preferring it over in-person visits.^37,46,66,67^ Other studies in this review indicated that patient satisfaction extends beyond interactions between patients and health care professionals and includes perceptions of overall care and treatment decisions.^46,64,66^ None of the reviewed articles specifically examined patient satisfaction among patients who do not have regular contact with health systems (i.e., they are not established patients of either primary care or specialty care facilities). In a survey of African American patients with hypertension in Mississippi,^46^ 97% of respondents reported receiving the care they needed through telehealth during the COVID-19 pandemic. Most respondents (81%) also said that the quality of care for telehealth visits was the same or better as in-person visits. In addition, there were some benefits related to patient engagement and knowledge.

Idris et al.^10^ found that telehealth appointments as a follow-up to data transmitted through RPM allowed for review of submitted data, monitoring of compliance with medication and diet, and patient education. These appointments led to patients feeling more in control of their health and gaining a better understanding of their conditions than in-person visits. This routine feedback, from an RPM or mHealth device along with feedback from health care professionals, can increase patient autonomy by helping them to understand the impact of behavioral decisions (e.g., diet, physical activity, and medication adherence) on managing their health condition.^68^

#### Telehealth during the COVID-19 pandemic

One study examined the effectiveness of telehealth on patient outcomes during the pandemic.^47^ In this study, authors affiliated with the telehealth company Doctors on Demand specifically examined the role of videoconferencing for hypertension management in the United States during the COVID-19 pandemic.^47^ The authors tracked videoconferencing among patients with diagnosed hypertension in their national database, who had more than two blood pressure readings during the study period (*N* = 569). Overall, 77% of the patients experienced an improvement in either SBP or DBP. Furthermore, among patients who completed satisfaction surveys (*N* = 508), ∼97% rated their visits as a 4 or 5 (out of a possible high score of 5 for satisfaction).

## Discussion

This literature review and meta-analysis identified the use of telehealth for a wide range of cardiovascular conditions, including hypertension, stroke, and heart failure. The most common applications of telehealth for cardiovascular conditions were RPM; teleconferencing and videoconference visits (including telestroke and home-based cardiac rehabilitation); and mHealth. Often, the interventions used one or more applications of telehealth.

The interventions ranged from the basic (e.g., bidirectional SMS text messaging between patient and health care professionals) to multicomponent interventions combining these various technologies (e.g., mHealth can include appointment and medication reminders through text messages and is paired with RPM). In the studies reviewed, patients receiving care through telehealth saw improved blood pressure and CVD-related outcomes, which were similar to, or better than, those receiving usual care. These findings were corroborated by our meta-analysis, which found no significant difference between telehealth and control groups for blood pressure changes and hospitalizations. In fact, there was a small, yet significant, reduction in all-cause mortality for patients receiving care through telehealth.

RPM was the most frequently reported intervention type found in this review and was used for monitoring both hypertension and heart failure patients. In conjunction with RPM, each of these studies included patient counseling (virtual or by phone) about the condition, challenges with treatment plans, and other related topics.

Below, we describe more fully four major findings, and their implications, from the review.

### RPM and its promise for hypertension and CVD outcomes

RPM significantly improved outcomes for mortality, yet its impacts on blood pressure and hospitalizations were not as pronounced. For studies that tested within-group differences, there were significant changes in blood pressure outcomes for patients using RPM. However, when comparing patients using RPM to those not using the technology, few studies found significant differences in blood pressure outcomes and hospitalization rates. This suggests that for managing blood pressure, RPM appears to be an effective complement to in-person care. There was also no significant difference in hospitalizations. Most of the studies in this review included a description of the protocol for abnormally high blood pressure values, which included having a patient transferred to the emergency department.

A 2020 systematic review of 91 RPM interventions for CVD in the international setting similarly reported mixed results for acute hospital use (i.e., hospitalizations, length of stay, emergency department presentation).^21^ RPM that involved an implantable device for the monitoring of CVD (i.e., invasive monitoring) effectively reduced hospital admissions compared to noninvasive monitoring, which suggests that the type of monitoring matters and was not distinguished in this meta-analysis.

A secondary analysis of the 2020 systematic review was conducted by Thomas et al. who qualitatively assessed the interventions and identified six factors that impact effectiveness of RPM interventions in acute care use.^22^ The identified factors include targeting populations at high risk, accurately detecting a decline in health, providing responsive and timely care, personalizing care, enhancing self-management, and ensuring collaborative and coordinated care, which offer a guided framework tailoring of future RPM interventions. Future studies can additionally examine the impact of RPM interventions on the timing of hospitalizations and intensity of treatment for patients with serious CVD-related conditions like heart failure.

To our knowledge, this literature review and meta-analysis is the first to summarize the impact of U.S.-based telehealth interventions on specific CVD and hypertension outcomes. The findings in our study are supported by systematic reviews on telehealth and CVD, but these reviews include evidence from other countries. For example, Safdar Khan et al.^20^ recently published a systematic global review of 20 telehealth and remote monitoring interventions for CVD between 2010 and 2020. Most of the studies (14 of 20) took place outside the United States.

The systematic review found “moderate-grade evidence of the beneficial effects of telehealth monitoring” among patients with CVD for all-cause mortality and hospital admissions. For example, the review noted reductions in all-cause mortality in telehealth interventions focused on patients with chronic heart failure in five of six studies—which originated in China (two studies), Belgium, Germany, Netherlands, and the United States. Snoswell et al.^23^ reviewed 24 meta-analyses of changes in mortality where telehealth health care interventions were delivered in multiple countries, including the United States, by five medical disciplines (cardiovascular, neurology, pulmonary, obstetrics, and intensive care). Significant reductions in all-cause mortality were observed in 8 of the 13 meta-analyses of patients with heart failure, who used telehealth, compared to patients engaged in usual care.

### Team-based approaches as a benefit of telehealth

Another potential benefit of telehealth for CVD and associated risk factors is that patients can connect with a variety of health care professionals to manage their condition, not just physicians. Most of the studies reviewed included nurses, pharmacists, and other clinical staff as first-line responders to patients. In several ways, telehealth supports the concept of team-based care delivery. For example, while physicians play a role in most RPM programs, they are not commonly led directly by physicians.

Most RPM interventions use a team-based approach with a nurse, dietician, and/or pharmacist forming the care team for the patient. Physicians are updated about their patients, typically through the EHR system. Similarly, home-based cardiac rehabilitation programs utilize a team comprising physicians, nurses, physiotherapists, and exercise physiologists. The fact that the American Society for Preventive Cardiology released a clinical guideline in 2020 in response to the pandemic recommending cardiovascular care teams expand care delivery using telehealth^69^ is promising, yet it remains to be seen how these guidelines will or will not change once COVID-19 enters an endemic phase in the Umited States.

This review suggests that patients having a method of communicating with health care professionals outside of a traditional care setting may be helpful, both to keep patients engaged and abreast of their health condition and to identify potential barriers to disease management. Several studies described how engaging patients through telehealth systems helped health care professionals to identify problems with medication effectiveness and adverse effects faster than the traditional in-office appointments.

Health care professionals received regular updates about a patient's health status, with vitals such as blood pressure and weight captured in regular intervals ranging from daily to weekly. These electronic interactions also helped health care professionals identify barriers to following the treatment protocol and intervening, as needed, working with a pharmacy to ensure refills were received in a timely manner. Thomas and colleagues' qualitative review of RPM interventions identified multidisciplinary team-based collaborative and coordinated care as a key mechanism for effective RPM interventions and offers further support for our finding.^22^

### Combination interventions as a promising model for care delivery

Half of the interventions in the review involved combination interventions, which points to an area worthy of further exploration, particularly around impacts of combination interventions on patient outcomes and satisfaction. For example, the combination of RPM plus mHealth was frequently mentioned in the articles reviewed, and the pairing of technologies may provide multiple avenues of communicating with patients, which could have tangible effects on patient clinical outcomes. This review also found that hybrid approaches—for example, the use of both remote and in-person visits following RPM for heart failure patients—may be worthy of future consideration, especially in light of potential challenges for patients to attend in-person follow-up visits.

### Implications for social determinants of health and health disparities: more exploration of telehealth strategies that advance health equity

A dearth of evidence exists describing the impact of telehealth on equitable CVD- and hypertension-related outcomes. The HINTS study described the effectiveness of telemonitoring on blood pressure outcomes among a diverse study population, but did not assess outcomes by patient characteristics,^42^ which highlights a major gap in the literature. In a follow-up to the original HINTS study, Jackson and colleagues^70^ examined whether there were different effects of the trial on African American and non-Hispanic White patients. African American patients receiving the combined intervention of medication and behavioral health management through teleconferencing and the use of blood pressure monitoring devices showed larger improvements in blood pressure control compared to the African American patients in the usual care group.

There was no significant difference between intervention groups for non-Hispanic White patients in the study, and no analysis comparing effects by race was reported. The only other available evidence related to health disparities was reported by researchers at NYU Grossman School of Medicine,^71^ who presented a novel study (not yet published) of Black and Hispanic stroke survivors with uncontrolled blood pressure at the International Stroke Conference 2020. The study found that patients who received home blood pressure monitoring plus telephone-based lifestyle counseling by nurses had greater reductions in SBP than patients who participated only in home blood pressure monitoring. The evidence for addressing health disparities through telehealth is mixed and still emerging.

The lack of peer-reviewed articles focused on addressing social determinants of health and health disparities through telehealth interventions for hypertension and CVD affects our ability to understand the impact of telehealth on populations that are disproportionately affected by such conditions. Advancing health equity through telehealth requires consideration of myriad factors, including socioeconomic status (e.g., education, income, occupation, employment status), health care access, geographic location, and policies, all of which can affect adoption of telehealth in health care systems.

Future studies can examine, among these populations most disproportionately affected by the factors, how telehealth is used, any unique barriers to utilization, and the impact of these factors on hypertension- and CVD-related outcomes. In addition, for interventions focusing on populations at highest risk, having comparison groups will help improve the strength of the evidence available, giving us an understanding of the intervention effects on those most at risk compared to those with lower risk. This work is an opportunity to develop evidence-based practices and clearly delineate the program components that lead to successful outcomes.

### Limitations

There are several potential limitations to our approach. First, there was a limited amount of peer-reviewed literature on clinically based telehealth interventions for hypertension and CVD in the United States. The review was limited in the number of studies that examined disproportionately affected populations, thereby preventing our ability to conduct additional subgroup analyses and thoroughly explore health equity implications.

For the meta-analyses, because our estimates of blood pressure and CVD-related outcomes were obtained by combining individual estimates for the published literature, the quality of estimates depended on the quality of underlying studies and our ability to harmonize the data. Variation in reporting format and outcome data provided by the study authors affected the number of studies included in the meta-analysis. Another limitation of the meta-analysis is that the hospitalization and mortality outcomes that were amenable to analysis in the meta-analysis were all cause rather than cause specific. The lack of literature that specifically reports the impact of telehealth interventions on CVD- or hypertension-related hospitalizations or mortality highlights a major gap in the literature.

Finally, publication bias, or the fact that studies with statistically significant results are more likely to be published than studies without statistically significant results, is a common concern with meta-analyses. As a result, our meta-analysis could have underrepresented studies with negative/null findings.

## Conclusion

In summary, the evidence presented in this study has shown that telehealth interventions are a successful complement to in-person care for hypertension- and CVD-related outcomes such as changes in blood pressure control, hospitalizations, and mortality for some populations and specific intervention approaches. Across all outcomes among the literature included, the evidence supported telehealth as a similar or complementary strategy to in-person methods. These findings demonstrate telehealth as a comparable method for helping some patients with hypertension and/or CVD manage their conditions. Through these interventions, health care professionals can be more responsive to the needs of patients and adapt treatment plans faster, resulting in improvements in medication adherence.

In addition, telehealth has also been shown to improve patient care and clinical outcomes through its impact on hospitalization and mortality. Outside of clinical outcomes, telehealth interventions appear to improve patient engagement and satisfaction with care, which can help empower patients to make more informed choices to manage their condition. However, further research and evaluation are needed to understand the impact of telehealth on equitable access to high-quality and affordable health care services for the control and management of hypertension and CVD, especially for patients most at risk for hypertension and CVD.

## Disclaimer

The opinions expressed by authors contributing to this journal do not necessarily reflect the opinions of the U.S. Department of Health and Human Services, the Public Health Service, the Centers for Disease Control and Prevention, or the authors' affiliated institutions. No copyrighted material was used in this research or this article.

## Abbreviations Used

BP: blood pressure
CPS: clinical pharmacy specialists
CDC: Centers for Disease Control and Prevention
COPD: chronic obstructive pulmonary disease
CVD: cardiovascular disease
DBP: diastolic blood pressure
ED: emergency department
EHR: electronic health record
HINTS: Hypertension Intervention Nurse Telemedicine Study
IVR: interactive voice response
mHealth: mobile health technologies
PRISMA: Preferred Reporting Items for Systematic Reviews and Meta-Analyses
RDNs: registered dietitians and nutritionists
RNs: registered nurses
RPM: Remote Patient Monitoring
SBP: systolic blood pressure

## References

[1] Centers for Disease Control and Prevention. About Heart Disease. 2022. Available from: https://www.cdc.gov/heartdisease/about.htm [Last accessed: March 22, 2022].

[2] Ahmad FB, Anderson RN. The leading causes of death in the US for 2020. JAMA 2021;325(18):1829–1830; doi: 10.1001/jama.2021.5469 33787821 PMC8145781

[3] Centers for Disease Control and Prevention. Facts about Hypertension. 2022. Available from: https://www.cdc.gov/bloodpressure/facts.htm [Last accessed: January 22, 2022].

[4] Centers for Disease Control and Prevention. Hypertension Cascade: Hypertension Prevalence, Treatment and Control Estimates among U.S. Adults Aged 18 Years and Older Applying the Criteria from the American College of Cardiology and American Heart Association's 2017 Hypertension Guideline—NHANES 2015–2018. U.S. Department of Health and Human Services: Atlanta, GA; 2021.

[5] Fang J, Yang Q, Ayala C, et al. Disparities in access to care among US adults with self-reported hypertension. Am J Hypertens 2014;27(11):1377–1386; doi: 10.1093/ajh/hpu061 24847953 PMC4263941

[6] Centers for Disease Control and Prevention. Telehealth Interventions to Improve Chronic Disease. 2020. Available from: https://www.cdc.gov/dhdsp/pubs/telehealth.htm [Last accessed: March 4, 2022].

[7] Gandapur Y, Kianoush S, Kelli HM, et al. The role of mHealth for improving medication adherence in patients with cardiovascular disease: a systematic review. Eur Heart J Qual Care Clin Outcomes 2016;2(4):237–244; doi: 10.1093/ehjqcco/qcw018 29474713 PMC5862021

[8] Angellotti E, Wong JB, Pierce A, et al. Combining Wireless Technology and Behavioral Economics to Engage Patients (WiBEEP) with cardiometabolic disease: A pilot study. Pilot Feasibility Stud 2019;5:7; doi: 10.1186/s40814-019-0395-8 30675374 PMC6332845

[9] Fisher NDL, Fera LE, Dunning JR, et al. Development of an entirely remote, non-physician led hypertension management program. Clin Cardiol 2019;42(2):285–291; doi: 10.1002/clc.23141 30582181 PMC6712321

[10] Idris S, Degheim G, Ghalayini W, et al. Home telemedicine in heart failure: A pilot study of integrated telemonitoring and virtual provider appointments. Rev Cardiovasc Med 2015;16(2):156–162. 26198562 10.3909/ricm0760

[11] Kao DP, Lindenfeld J, Macaulay D, et al. Impact of a telehealth and care management program on all-cause mortality and healthcare utilization in patients with heart failure. Telemed J E Health 2016;22(1):2–11; doi: 10.1089/tmj.2015.0007 26218252 PMC4739127

[12] Maciejewski ML, Bosworth HB, Olsen MK, et al. Do the benefits of participation in a hypertension self-management trial persist after patients resume usual care? Circ Cardiovasc Qual Outcomes 2014;7(2):269–275; doi: 10.1161/CIRCOUTCOMES.113.000309 24619321

[13] Margolis KL, Asche SE, Bergdall AR, et al. A successful multifaceted trial to improve hypertension control in primary care: Why did it work? J Gen Intern Med 2015;30(11):1665–1672; doi: 10.1007/s11606-015-3355-x 25952653 PMC4617923

[14] Milani RV, Wilt JK, Milani AR, et al. Digital management of hypertension improves systolic blood pressure variability. Am J Med 2020;133(7):e355–e359; doi: 10.1016/j.amjmed.2019.10.043 31870666

[15] Ralston JD, Cook AJ, Anderson ML, et al. Home blood pressure monitoring, secure electronic messaging and medication intensification for improving hypertension control: a mediation analysis. Appl Clin Inf 2014;5(1):232–248; doi: 10.4338/ACI-2013-10-RA-0079 PMC3974258 24734136

[16] Updike WH, Pane O, Hanna K, et al. Pharmacists interventions using Bluetooth technology and telehealth to improve blood pressure—A pilot study. J Am Pharm Assoc 2020;60(4):e100–e108; doi: 10.1016/j.japh.2020.01.008 32094040

[17] Magid DJ, Ho PM, Olson KL, et al. A multimodal blood pressure control intervention in 3 healthcare systems. Am J Manag Care 2011;17(4):e96–e103. 21774100

[18] Kruse CS, Soma M, Pulluri D, et al. The effectiveness of telemedicine in the management of chronic heart disease—A systematic review. JRSM Open 2017;8(3):205427041668174; doi: 10.1177/2054270416681747 PMC5347273 28321319

[19] Takahashi EA, Schwamm LH, Adeoye OM, et al. An overview of telehealth in the management of cardiovascular disease: A scientific statement from the American Heart Association. Circulation 2022;CIR.0000000000001107; doi: 10.1161/CIR.0000000000001107 PMC11441725 36373541

[20] Safdar Khan U, Gunasegaran B, Behanan AM, et al. Telemedicine and use of remote monitoring in cardiovascular disease: A systematic review. Arch Intern Med Res 2022;05(01); doi: 10.26502/aimr.0085

[21] Taylor ML, Thomas EE, Snoswell CL, et al. Does remote patient monitoring reduce acute care use? A systematic review. BMJ Open 2021;11(3):e040232; doi: 10.1136/bmjopen-2020-040232 PMC7929874 33653740

[22] Thomas EE, Taylor ML, Banbury A, et al. Factors influencing the effectiveness of remote patient monitoring interventions: A realist review. BMJ Open 2021;11(8):e051844; doi: 10.1136/bmjopen-2021-051844 PMC8388293 34433611

[23] Snoswell CL, Chelberg G, De Guzman KR, et al. The clinical effectiveness of telehealth: A systematic review of meta-analyses from 2010 to 2019. J Telemed Telecare 2021;1357633X2110229; doi: 10.1177/1357633X211022907 34184580

[24] Garfan S, Alamoodi AH, Zaidan BB, et al. Telehealth utilization during the Covid-19 pandemic: A systematic review. Comput Biol Med 2021;138:104878; doi: 10.1016/j.compbiomed.2021.104878 34592585 PMC8450049

[25] Anonymous. Best Practices for Cardiovascular Disease Prevention Programs. National Center for Chronic Disease Prevention and Health Promotion (U.S.); 2022; doi: 10.15620/cdc:122290

[26] Henry TA. Why Telehealth Visits Shouldn't Mean Skipping BP Measurement. 2021. Available from: https://www.ama-assn.org/delivering-care/hypertension/why-telehealth-visits-shouldn-t-mean-skipping-bp-measurement [Last accessed: January 22, 2022].

[27] Bress AP, Cohen JB, Anstey DE, et al. Inequities in hypertension control in the United States exposed and exacerbated by COVID-19 and the role of home blood pressure and virtual health care during and After the COVID-19 pandemic. J Am Heart Assoc 2021;10(11):e020997; doi: 10.1161/JAHA.121.020997 34006116 PMC8483507

[28] Shea CM, Turner K, Alishahi Tabriz A, et al. Implementation strategies for telestroke: A qualitative study of telestroke networks in North Carolina. Telemed J E Health 2019;25(8):708–716; doi: 10.1089/tmj.2018.0131 30192206 PMC6684023

[29] Lin C-CC, Dievler A, Robbins C, et al. Telehealth in health centers: Key adoption factors, barriers, and opportunities. Health Aff (Millwood) 2018;37(12):1967–1974; doi: 10.1377/hlthaff.2018.05125 30633683

[30] StataCorp LP. Stata Statistical Software. 2019. Available from: https://www.stata.com/

[31] DerSimonian R, Laird N. Meta-analysis in clinical trials. Control Clin Trials 1986;7(3):177–188; doi: 10.1016/0197-2456(86)90046-2 3802833

[32] DerSimonian R, Laird N. Meta-analysis in clinical trials revisited. Contemp Clin Trials 2015;45(Pt A):139–145; doi: 10.1016/j.cct.2015.09.002 26343745 PMC4639420

[33] Choudhry NK, Isaac T, Lauffenburger JC, et al. Effect of a remotely delivered tailored multicomponent approach to enhance medication taking for patients with hyperlipidemia, hypertension, and diabetes: The STIC2IT Cluster Randomized Clinical Trial. JAMA Intern Med 2018;178(9):1182–1189; doi: 10.1001/jamainternmed.2018.3189 30083727 PMC6142966

[34] Mallow JA, Theeke LA, Theeke E, et al. The effectiveness of mI SMART: A nurse practitioner led technology intervention for multiple chronic conditions in primary care. Int J Nurs Sci 2018;5(2):131–137; doi: 10.1016/j.ijnss.2018.03.009 31406814 PMC6626240

[35] Bekelman DB, Plomondon ME, Carey EP, et al. Primary Results of the Patient-Centered Disease Management (PCDM) for heart failure study: A randomized clinical trial. JAMA Intern Med 2015;175(5):725–732; doi: 10.1001/jamainternmed.2015.0315 25822284

[36] Benson GA, Sidebottom A, Sillah A, et al. Reach and effectiveness of the HeartBeat Connections telemedicine pilot program. J Telemed Telecare 2018;24(3):216–223; doi: 10.1177/1357633X17692723 29278986

[37] Davis C, Bender M, Smith T, et al. Feasibility and acute care utilization outcomes of a post-acute Transitional Telemonitoring Program for underserved chronic disease patients. Telemed J E Health 2015;21(9):705–713; doi: 10.1089/tmj.2014.0181 25955129

[38] Margolis KL, Asche SE, Bergdall AR, et al. Effect of home blood pressure telemonitoring and pharmacist management on blood pressure control: A cluster randomized clinical trial. JAMA 2013;310(1):46–56; doi: 10.1001/jama.2013.6549 23821088 PMC4311883

[39] O'Connor M, Asdornwised U, Dempsey ML, et al. Using telehealth to reduce all-cause 30-day hospital readmissions among heart failure patients receiving skilled home health services. Appl Clin Inf 2016;7(2):238–247; doi: 10.4338/ACI-2015-11-SOA-0157 PMC4941836 27437037

[40] Pekmezaris R, Mitzner I, Pecinka KR, et al. The impact of remote patient monitoring (telehealth) upon Medicare beneficiaries with heart failure. Telemed J E Health 2012;18(2):101–108; doi: 10.1089/tmj.2011.0095 22283360

[41] Rosen D, McCall JD, Primack BA. Telehealth protocol to prevent readmission among high-risk patients with congestive heart failure. Am J Med 2017;130(11):1326–1330; doi: 10.1016/j.amjmed.2017.07.007 28756266

[42] Bosworth HB, Powers BJ, Olsen MK, et al. Home blood pressure management and improved blood pressure control: Results from a randomized controlled trial. Arch Intern Med 2011;171(13):1173–1180; doi: 10.1001/archinternmed.2011.276 21747013

[43] Wakefield BJ, Holman JE, Ray A, et al. Outcomes of a home telehealth intervention for patients with diabetes and hypertension. Telemed E Health 2012;18(8):575–579; doi: 10.1089/tmj.2011.0237 22873700

[44] Friedberg JP, Robinaugh DJ, Wang B, et al. Who is being reached for a telephone-delivered intervention for patients with uncontrolled hypertension? Telemed J E Health 2014;20(3):229–234; doi: 10.1089/tmj.2013.0071 24386927

[45] Litke J, Spoutz L, Ahlstrom D, et al. Impact of the clinical pharmacy specialist in telehealth primary care. Am J Health Syst Pharm 2018;75(13):982–986; doi: 10.2146/ajhp170633 29941537

[46] Mills KT, Peacock E, Chen J, et al. Experiences and beliefs of low-income patients with hypertension in Louisiana and Mississippi during the COVID-19 pandemic. J Am Heart Assoc 2021;10(3):e018510; doi: 10.1161/JAHA.120.018510 33267723 PMC7955429

[47] Taylor P, Berg C, Thompson J, et al. Effective access to care in a crisis period: Hypertension control during the COVID-19 pandemic via telemedicine. Mayo Clin Proc Innov Qual Outcomes 2021;6(1):19–26; doi: 10.1016/j.mayocpiqo.2021.11.006 34805763 PMC8590930

[48] Baidwan NK, Davlyatov G, Mehta T. Telehealth use among community health centers and cardio-metabolic health outcomes. Healthcare (Basel) 2020;8(2):165; doi: 10.3390/healthcare8020165 32532120 PMC7348805

[49] Dalouk K, Gandhi N, Jessel P, et al. Outcomes of telemedicine video-conferencing clinic versus in-person clinic follow-up for implantable cardioverter-defibrillator recipients. Circ Arrhythm Electrophysiol 2017;10(9):e005217; doi: 10.1161/CIRCEP.117.005217 28916510

[50] Yuan N, Botting PG, Elad Y, et al. Practice patterns and patient outcomes after widespread adoption of remote heart failure care. Circ Heart Fail 2021;14(10):e008573; doi: 10.1161/CIRCHEARTFAILURE.121.008573 34587763 PMC8530957

[51] de Peralta S, Ziaeian B, Chang D, et al. Leveraging telemedicine for management of veterans with heart failure during COVID-19. J Am Assoc Nurse Pract 2021;34(1):182–187; doi: 10.1097/JXX.0000000000000573 33625164 PMC8371074

[52] Sobhani F, Desai S, Madill E, et al. Remote longitudinal inpatient acute stroke care via telestroke. J Stroke Cerebrovasc Dis 2021;30(6):105749; doi: 10.1016/j.jstrokecerebrovasdis.2021.105749 33784522

[53] Simpson AN, Harvey JB, DiLembo SM, et al. Population health indicators associated with a Statewide Telestroke Program. Telemed J E Health 2020;26(9):1126–1133; doi: 10.1089/tmj.2019.0204 32045330 PMC7482719

[54] Dumitrascu O, Demaerschalk B. Telestroke. Curr Cardiol Rep 2017;19(9):85; doi: 10.1007/s11886-017-0895-1 28785990

[55] Ovbiagele B, Jenkins C, Patel S, et al. Mobile health medication adherence and blood pressure control in recent stroke patients. J Neurol Sci 2015;358(1–2):535–537; doi: 10.1016/j.jns.2015.10.008 26463572

[56] Piette JD, Striplin D, Marinec N, et al. A mobile health intervention supporting heart failure patients and their informal caregivers: A randomized comparative effectiveness trial. J Med Internet Res 2015;17(6):e142; doi: 10.2196/jmir.4550 26063161 PMC4526929

[57] Polgreen LA, Carter BL, Polgreen PM, et al. A pharmacist intervention for monitoring and treating hypertension using bidirectional texting: PharmText BP. Contemp Clin Trials 2020;98:106169; doi: 10.1016/j.cct.2020.106169 33038500 PMC7686143

[58] Zha P, Qureshi R, Porter S, et al. Utilizing a mobile health intervention to manage hypertension in an underserved community. West J Nurs Res 2020;42(3):201–209; doi: 10.1177/0193945919847937 31057081

[59] Clark HR, Goyder E, Bissell P, et al. How do parents' child-feeding behaviours influence child weight? Implications for childhood obesity policy. J Public Health 2007;29(2):132–141; doi: 10.1093/pubmed/fdm012 17442696

[60] Kothapalli P, Bove AA, Santamore WP, et al. Factors affecting frequency of patient use of internet-based telemedicine to manage cardiovascular disease risk. J Telemed Telecare 2013;19(4):205–208; doi: 10.1177/1357633x13487101 23666439

[61] Lakshminarayan K, Westberg S, Northuis C, et al. A mHealth-based care model for improving hypertension control in stroke survivors: Pilot RCT. Contemp Clin Trials 2018;70:24–34; doi: 10.1016/j.cct.2018.05.005 29763657 PMC6317360

[62] Corbett JA, Opladen JM, Bisognano JD. Telemedicine can revolutionize the treatment of chronic disease. Int J Cardiol Hypertens 2020;7:100051; doi: 10.1016/j.ijchy.2020.100051 33330846 PMC7490579

[63] Blum K, Gottlieb SS. The effect of a randomized trial of home telemonitoring on medical costs, 30-day readmissions, mortality, and health-related quality of life in a cohort of community-dwelling heart failure patients. J Card Fail 2014;20(7):513–521; doi: 10.1016/j.cardfail.2014.04.016 24769270

[64] Bowles KH, Hanlon AL, Glick HA, et al. Clinical effectiveness, access to, and satisfaction with care using a telehomecare substitution intervention: A randomized controlled trial. Int J Telemed Appl 2011;2011:540138; doi: 10.1155/2011/540138 22187551 PMC3236461

[65] Ong MK, Romano PS, Edgington S, et al. Effectiveness of remote patient monitoring after discharge of hospitalized patients with heart failure: The Better Effectiveness After Transition—Heart Failure (BEAT-HF) randomized clinical trial. JAMA Intern Med 2016;176(3):310–318; doi: 10.1001/jamainternmed.2015.7712 26857383 PMC4827701

[66] Karimi M, Lee EC, Couture SJ, et al. National Trends in Telehealth Use in 2021: Disparities in Utilization and Audio vs. Video Services. Office of the Assistant Secretary for Planning and Evaluation, U.S. Department of Health and Human Services: Washington, DC; 2022.

[67] Donelan K, Barreto EA, Sossong S, et al. Patient and clinician experiences with telehealth for patient follow-up care. Am J Manag Care 2019;25(1):40–44. 30667610

[68] Thangada ND, Garg N, Pandey A, et al. The emerging role of mobile-health applications in the management of hypertension. Curr Cardiol Rep 2018;20(9):78; doi: 10.1007/s11886-018-1022-7 30046971

[69] Khera A, Baum SJ, Gluckman TJ, et al. Continuity of care and outpatient management for patients with and at high risk for cardiovascular disease during the COVID-19 pandemic: A scientific statement from the American Society for Preventive Cardiology. Am J Prev Cardiol 2020;1:100009; doi: 10.1016/j.ajpc.2020.100009 32835347 PMC7194073

[70] Jackson GL, Oddone EZ, Olsen MK, et al. Racial differences in the effect of a telephone-delivered hypertension disease management program. J Gen Intern Med 2012;27(12):1682–1689; doi: 10.1007/s11606-012-2138-x[Lastaccessed:March5,2022]. 22865016 PMC3509293

[71] Ogedegbe G. Telemonitoring plus Phone Counseling Lowers Blood Pressure among Black and Hispanic Stroke Survivors. 2022. Available from: https://newsroom.heart.org/news/telemonitoring-plus-phone-counseling-lowers-blood-pressure-among-black-and-hispanic-stroke-survivors [Last accessed: March 5, 2022].

